# Supplementary figures and images for: The S2 subunit of spike encodes diverse targets for functional antibody responses to SARS-CoV-2
Source: PLoS Pathog. 2024 Aug 2;20(8):e1012383. doi: 10.1371/journal.ppat.1012383 (PMC11324185; doi:10.1371/journal.ppat.1012383)

**A**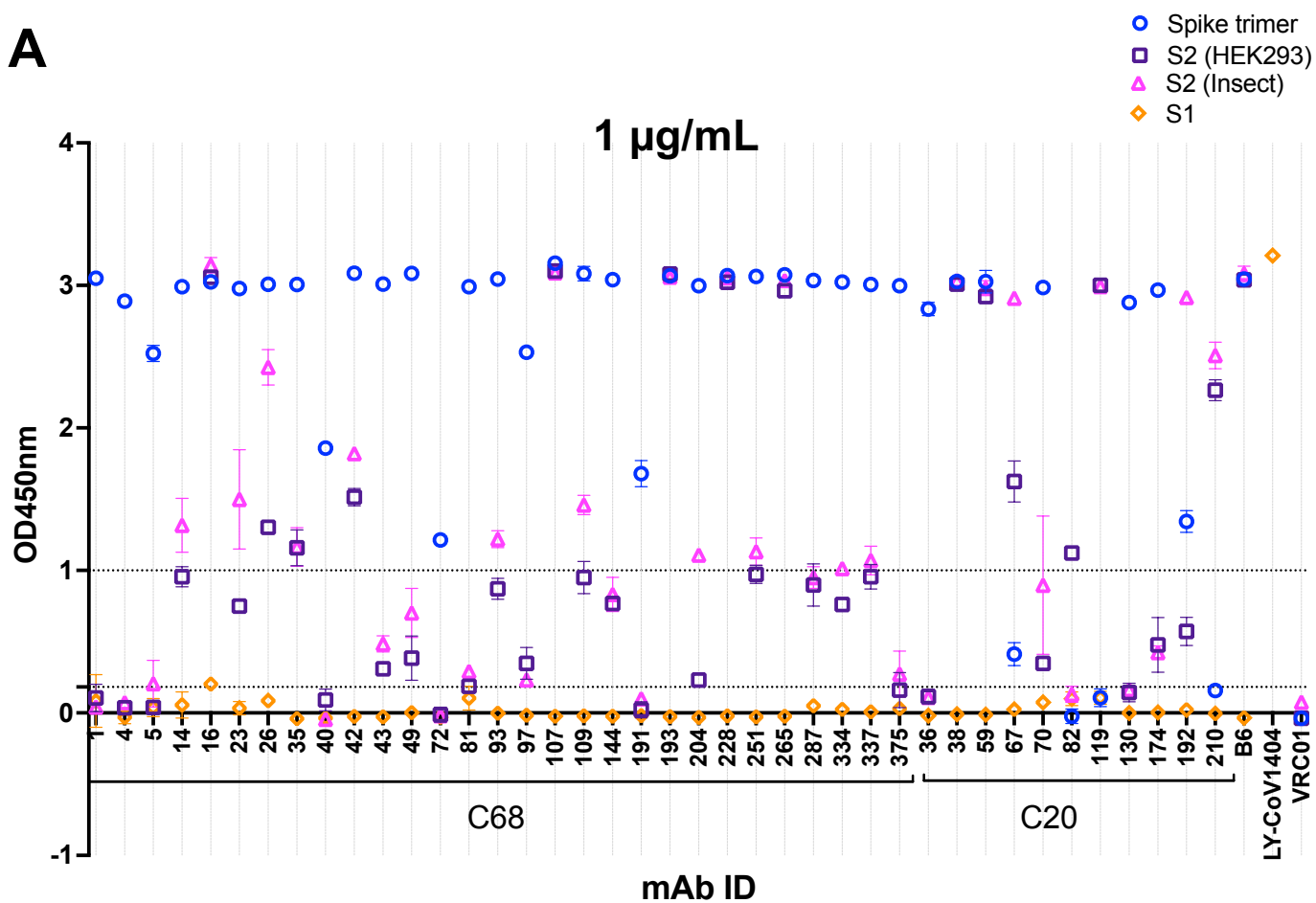**B**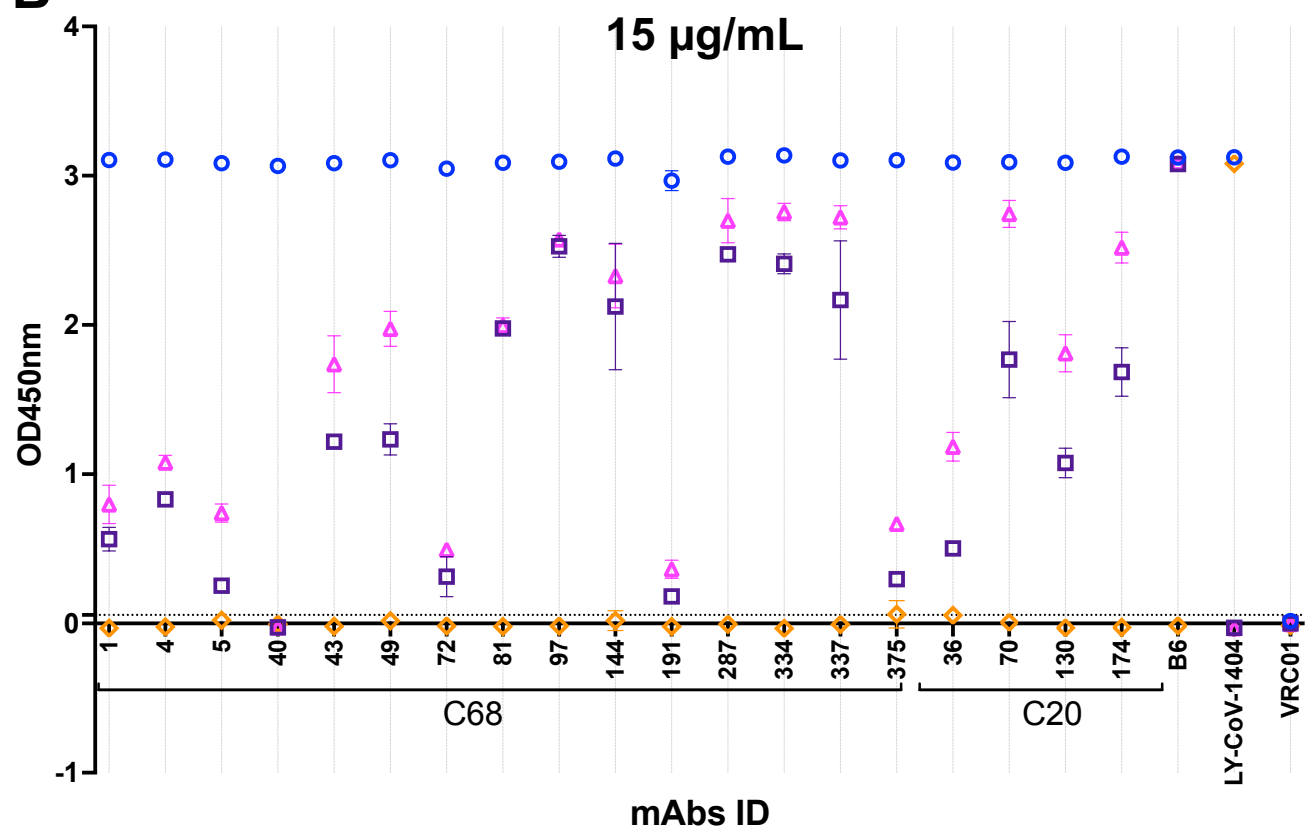

Supplement: S1 Fig — Binding of C68 and C20 mAbs at 1 μg/mL (A) or 15 μg/mL (B) to WH-1 spike trimer (blue circles), S2 protein produced in mammalian HEK293T cells (purple squares), S2 protein produced in insect cells (pink triangles), and S1 protein (orange triangles) by ELISA. Background-corrected OD450nm measures averaged across two technical replicates is shown. Control mAbs included CV3-25 and B6 (S2), LYCoV-1404 (S1/RBD), VRC01 (HIV mAb, negative control). Lower dashed lines represent the OD450nmave of VRC01 + 3 standard deviations to indicate measures above background. The dashed line at OD450nm = 1 in (A) identifies mAbs with binding values for S2 proteins < 1.0 that were tested at the higher concentration in (B). (PDF) [file ppat.1012383.s001.pdf]

**A**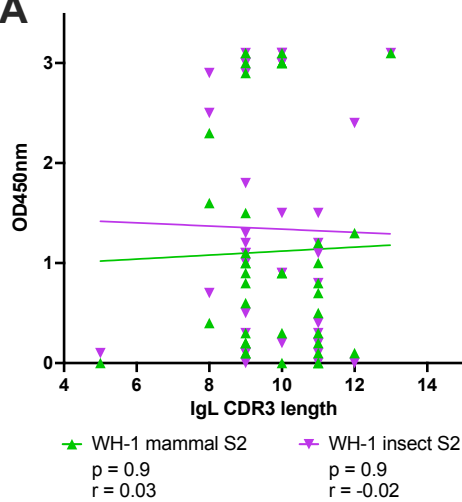**B**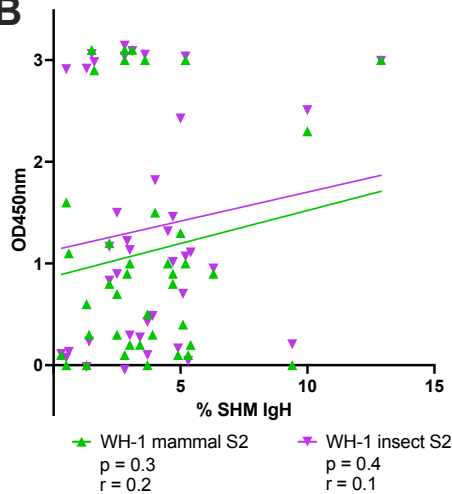**C**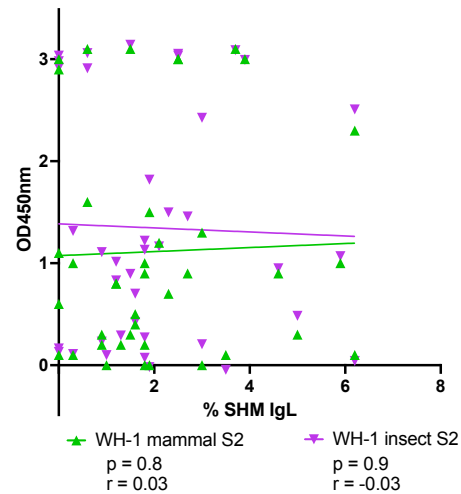**D**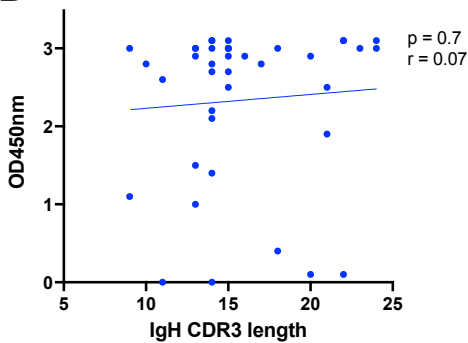**E**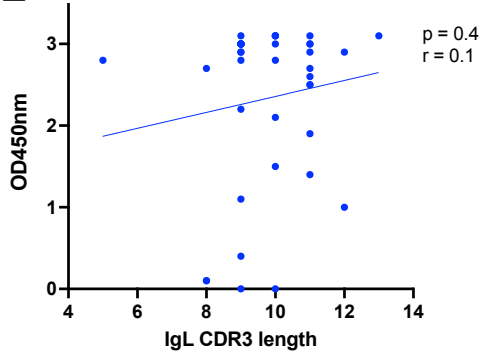**F**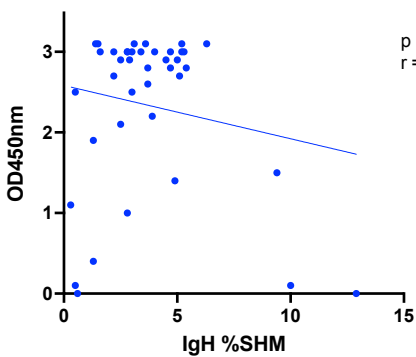**G**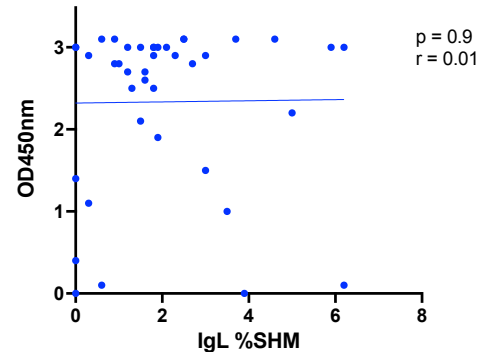

Supplement: S2 Fig — Correlation of IgL CDR3 length (A), IgH %SHM (B), IgL %SHM (C) to binding of S2 mAbs to WH-1 S2 protein produced in mammalian cells (green) or insect cells (purple). Correlation of IgH CDR3 length (D), IgL CDR3 length (E), IgH %SHM (F), IgL %SHM (G) to binding of S2 mAbs to WH-1 spike trimer (blue). All binding values from ELISAs (OD450nm) are averages of technical duplicates. Pearson’s correlation analysis was used to determine the strength of the correlation. (PDF) [file ppat.1012383.s002.pdf]

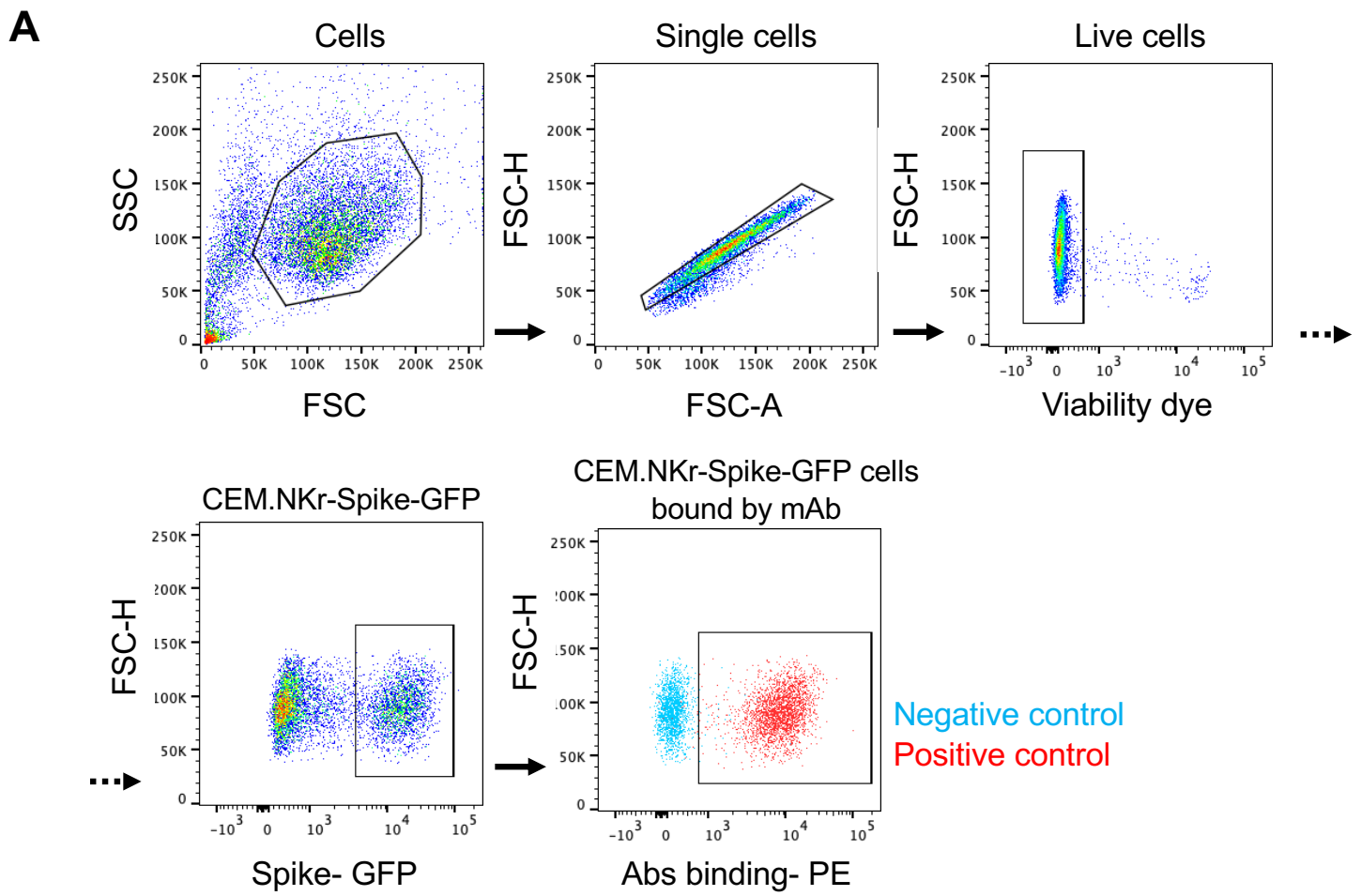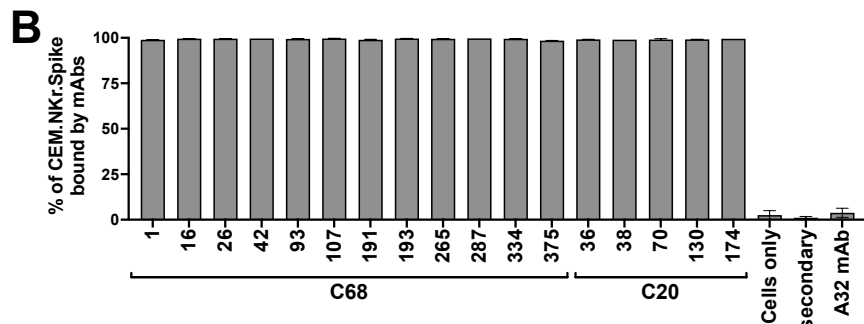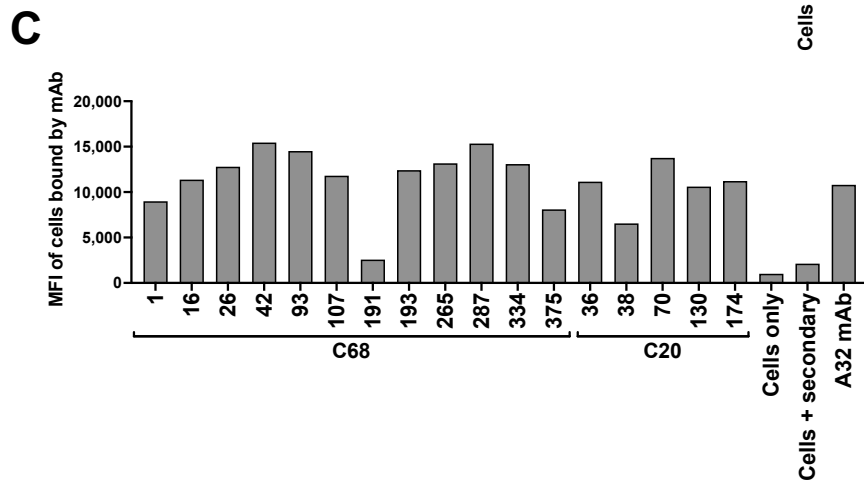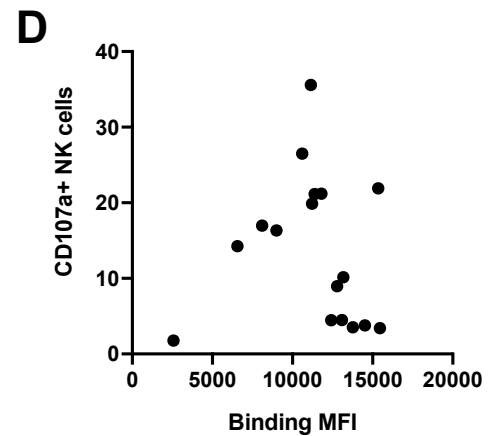

Supplement: S3 Fig — CEM.NKr CCR5+ cells expressing GFP-tagged D614G spike (CEM.NKr.Spike), which were used as target cells in the ADCC assays, bound by S2 mAbs identified by flow cytometry. The gating strategy is shown in (A). The % of CEM.NKr.Spike cells bound by mAbs (B) and the mean fluorescence intensity (MFI) of cells bound by antibody (C) are shown across the mAbs and controls: CEM.NKr.Spike cells without mAbs (cells only), CEM.NKr.Spike cells with secondary antibody (cells + secondary), CEM.NKr.Spike cells incubated with HIV mAb A32. (D) Correlation plot of the MFI of mAb bound CEM.NKr.Spike cells to CD107a+ natural killer (NK) cells. Pearson’s correlation analysis showed no association between the values (r = -0.6, p = 0.8). (PDF) [file ppat.1012383.s003.pdf]

**A**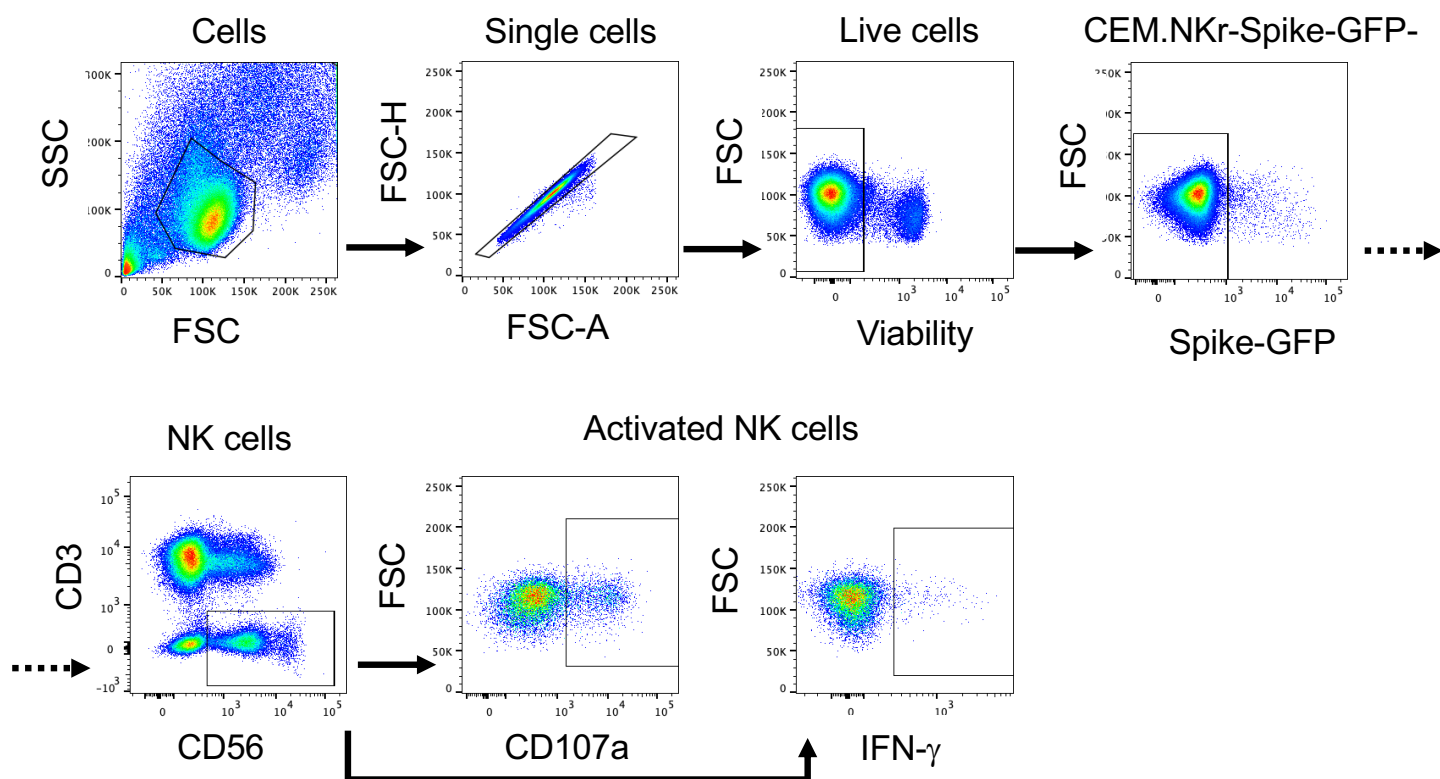**B**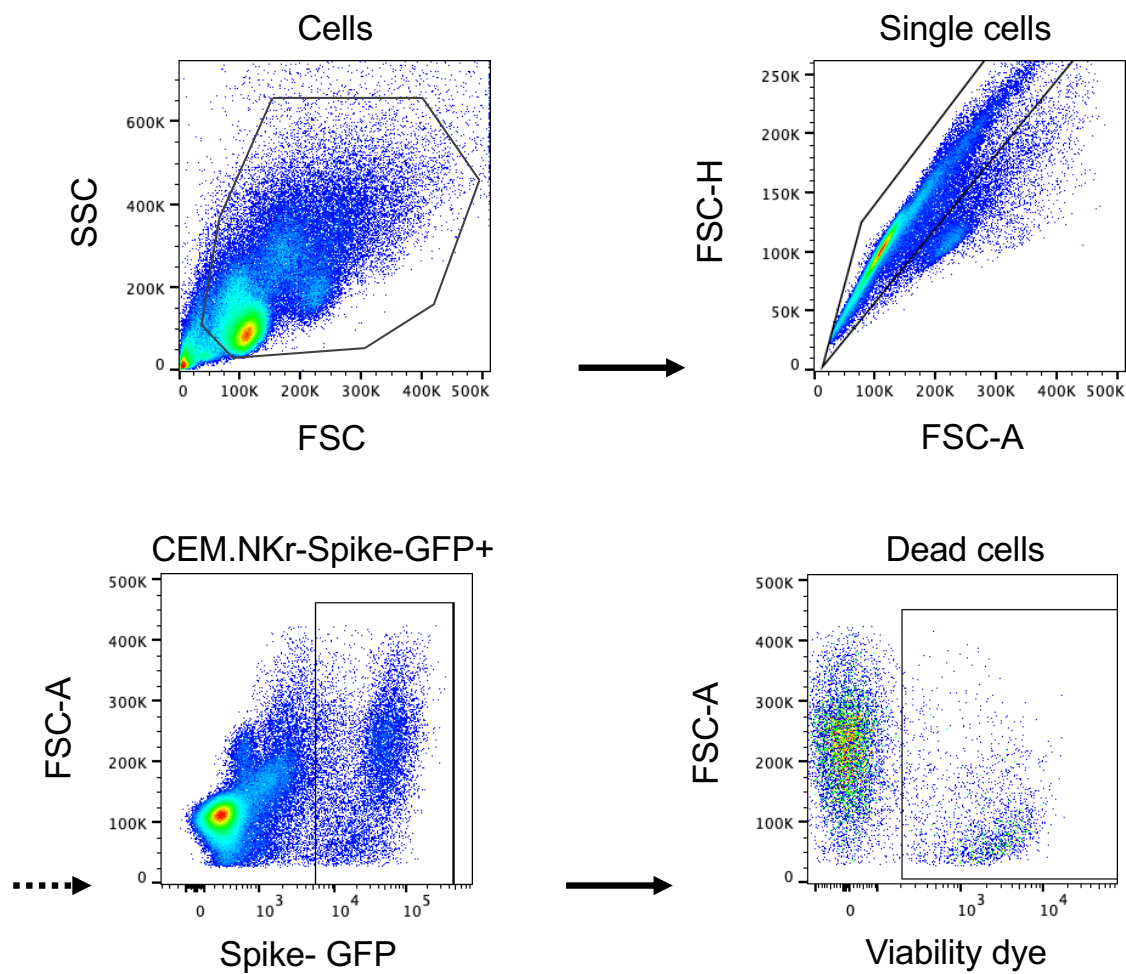

Supplement: S4 Fig — Identification of activated NK cells (CD107a+ or IFN-γ+) (A), and CEM.NKr.Spike cell death (B). (PDF) [file ppat.1012383.s004.pdf]

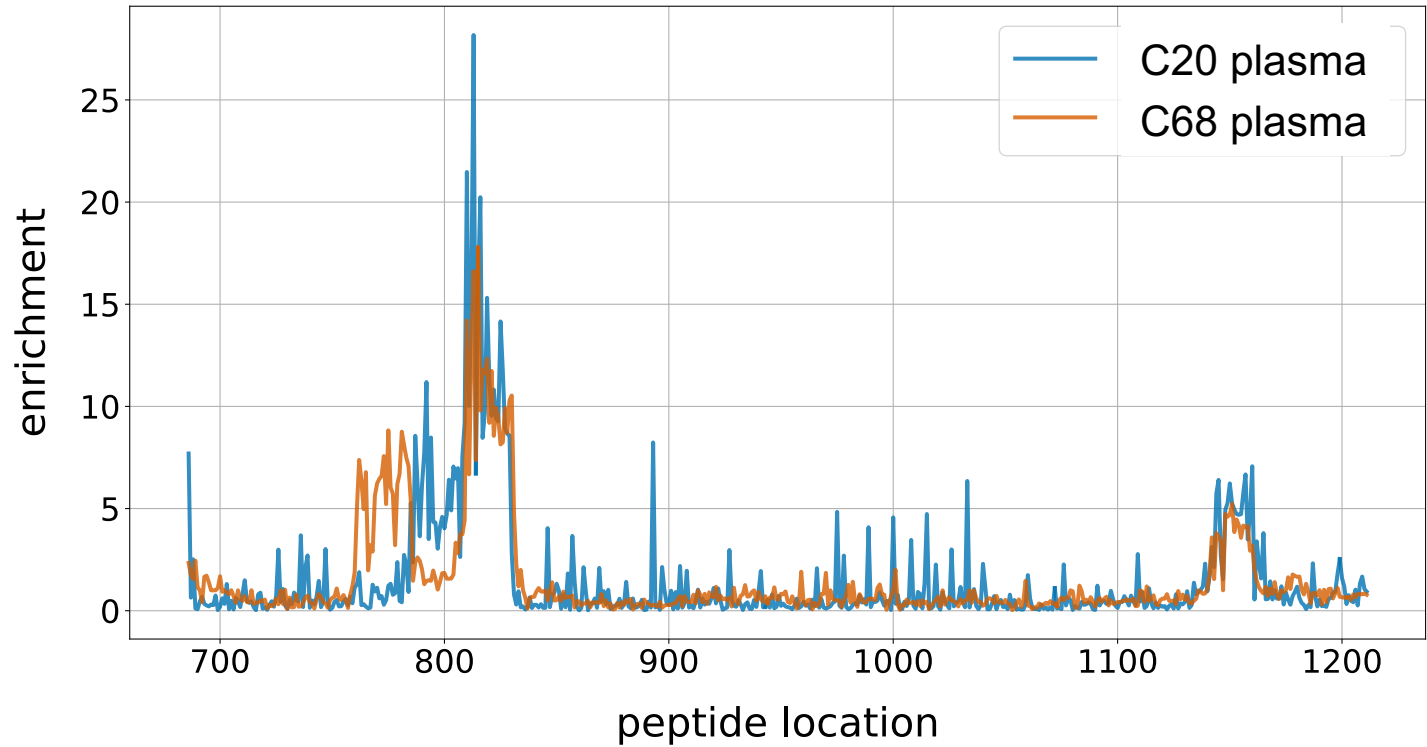

Supplement: S7 Fig — (PDF) [file ppat.1012383.s007.pdf]

**A****SARS-CoV-2 Spike**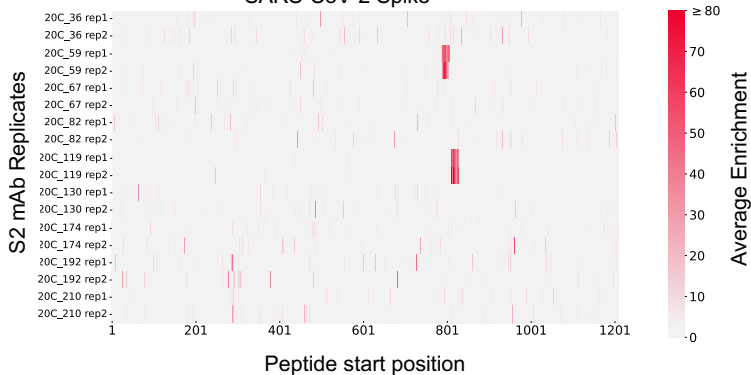**B****SARS-CoV-2 Spike**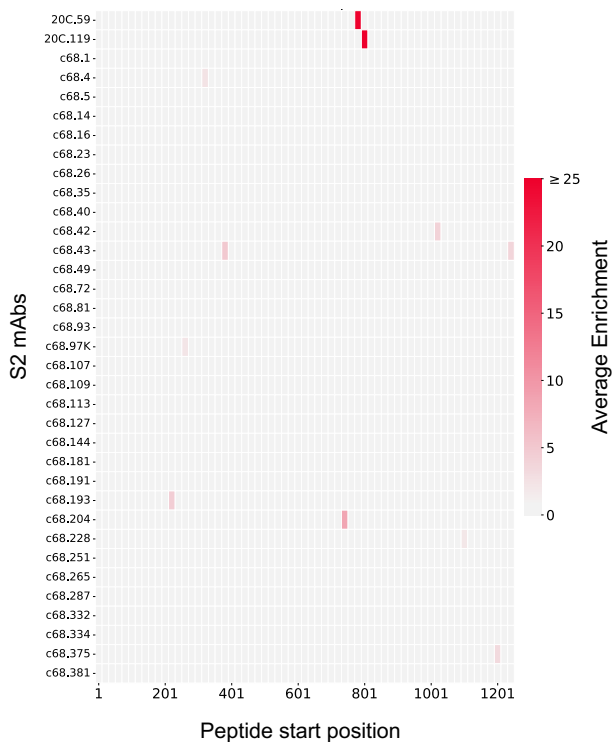

Supplement: S8 Fig — (A) Peptides enriched in library 1 (“Phage-DMS”) with C20 mAbs (technical replicates shown as separate rows). (B) Peptides enriched in library 2 (“pan-Cov”) with C68 mAbs (technical replicates averaged and regions at p<0.05 are shown. The scale of the enrichment is show by the red coloring with darker red meaning greater enrichment. (PDF) [file ppat.1012383.s008.pdf]

**A**

CV3-25

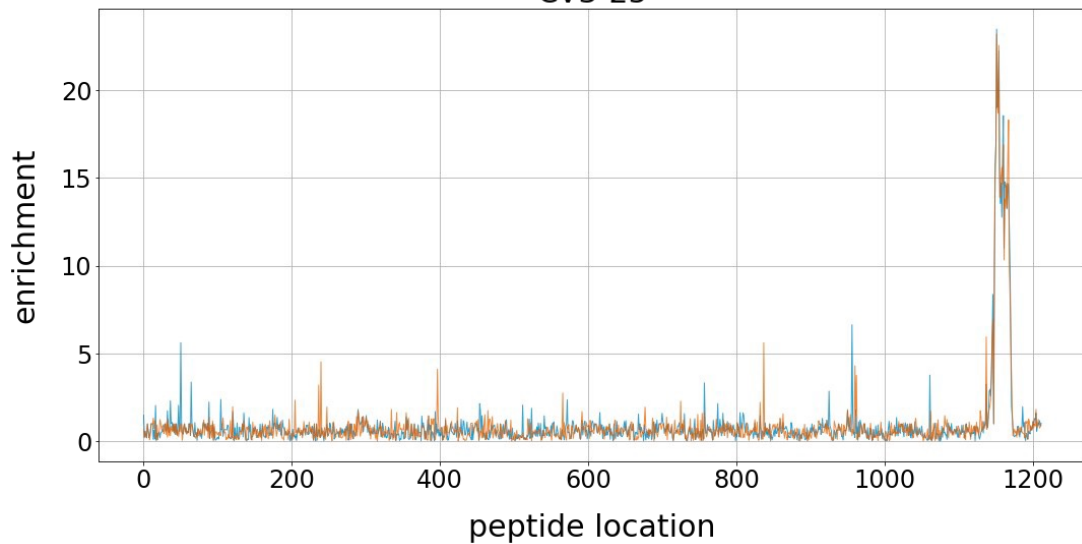**B**

1A9

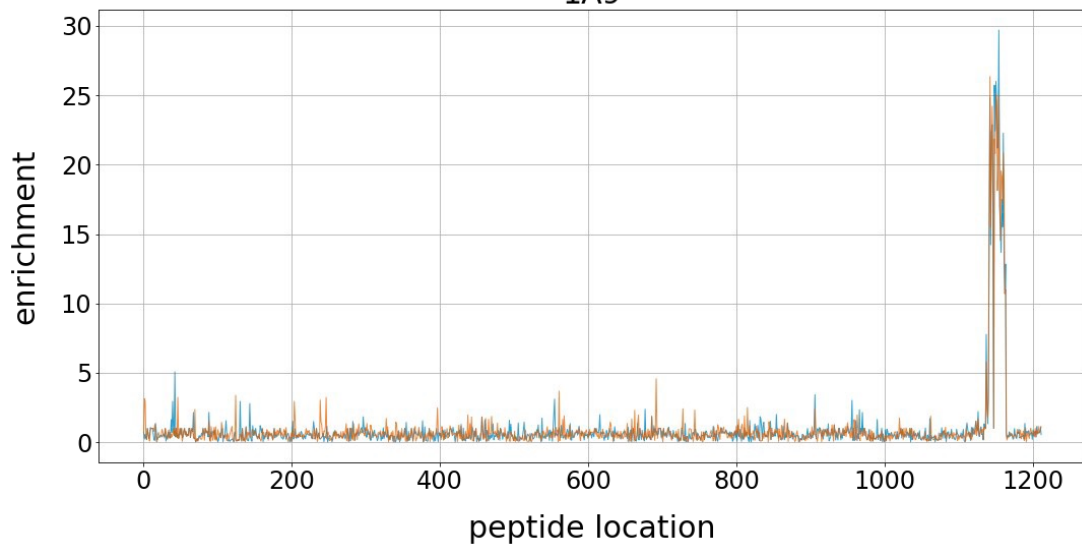

Supplement: S9 Fig — These mAbs were incubated with the Phage-DMS library and technical replicates are shown by the orange and blue graphs. Strong enrichment is shown for peptides in the SH region (1140–1160 aa) as expected [47,102]. (PDF) [file ppat.1012383.s009.pdf]

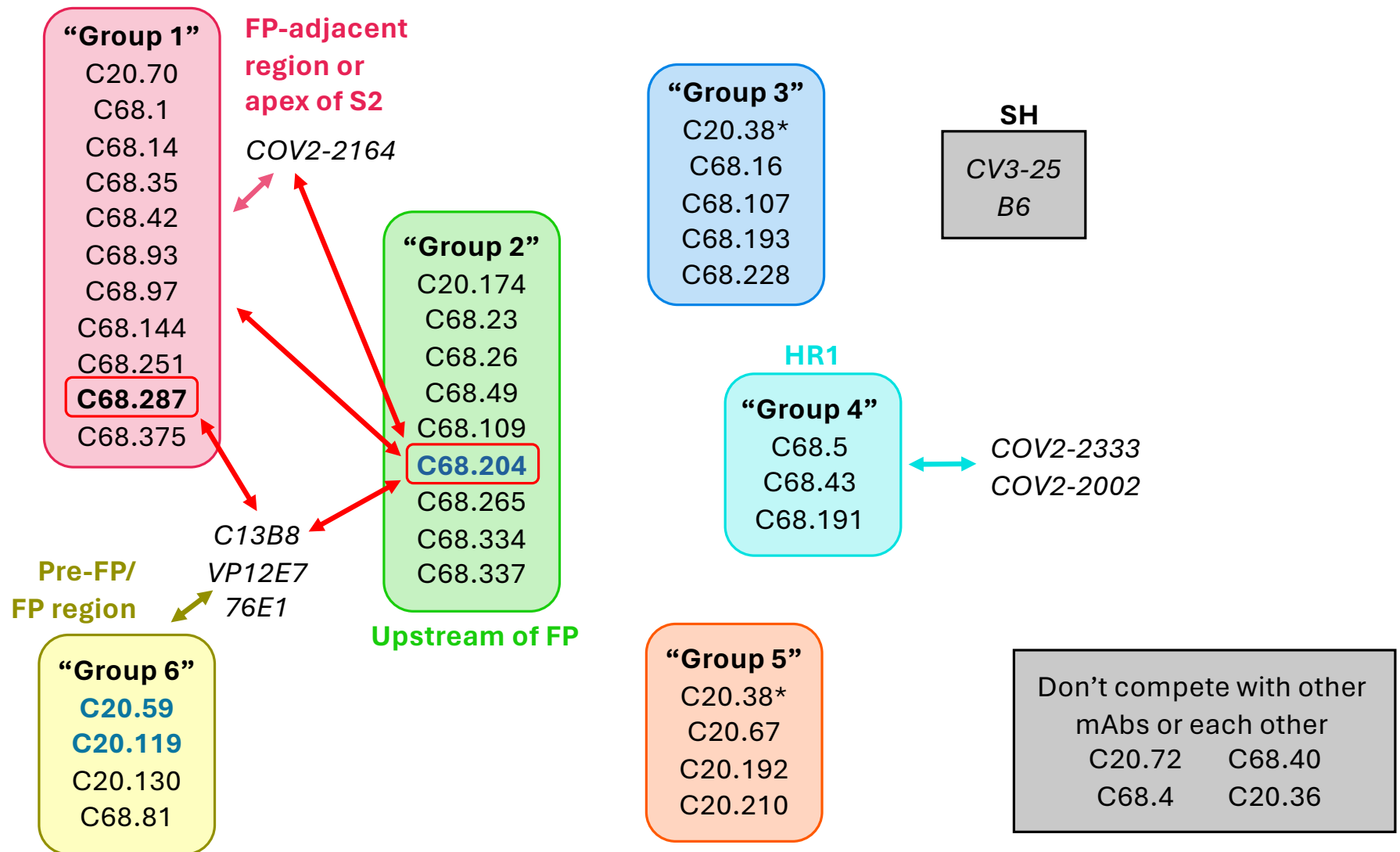

Supplement: S11 Fig — Binding competition groups of mAbs, as described and labeled in Fig 5, are shown. Reference antibodies with reported epitopes are in italics with the competition noted by the arrows. Predicted epitope regions for each group are noted in bold. MAbs that bound linear peptides in PhIP-seq are written in blue text. C68.287 and C68.204 compete with other mAbs outside of their groups and those interactions are noted in the red arrows. *C20.38 competed with two different groups depending on the antigen used and is listed in both groups in this diagram. (PDF) [file ppat.1012383.s011.pdf]

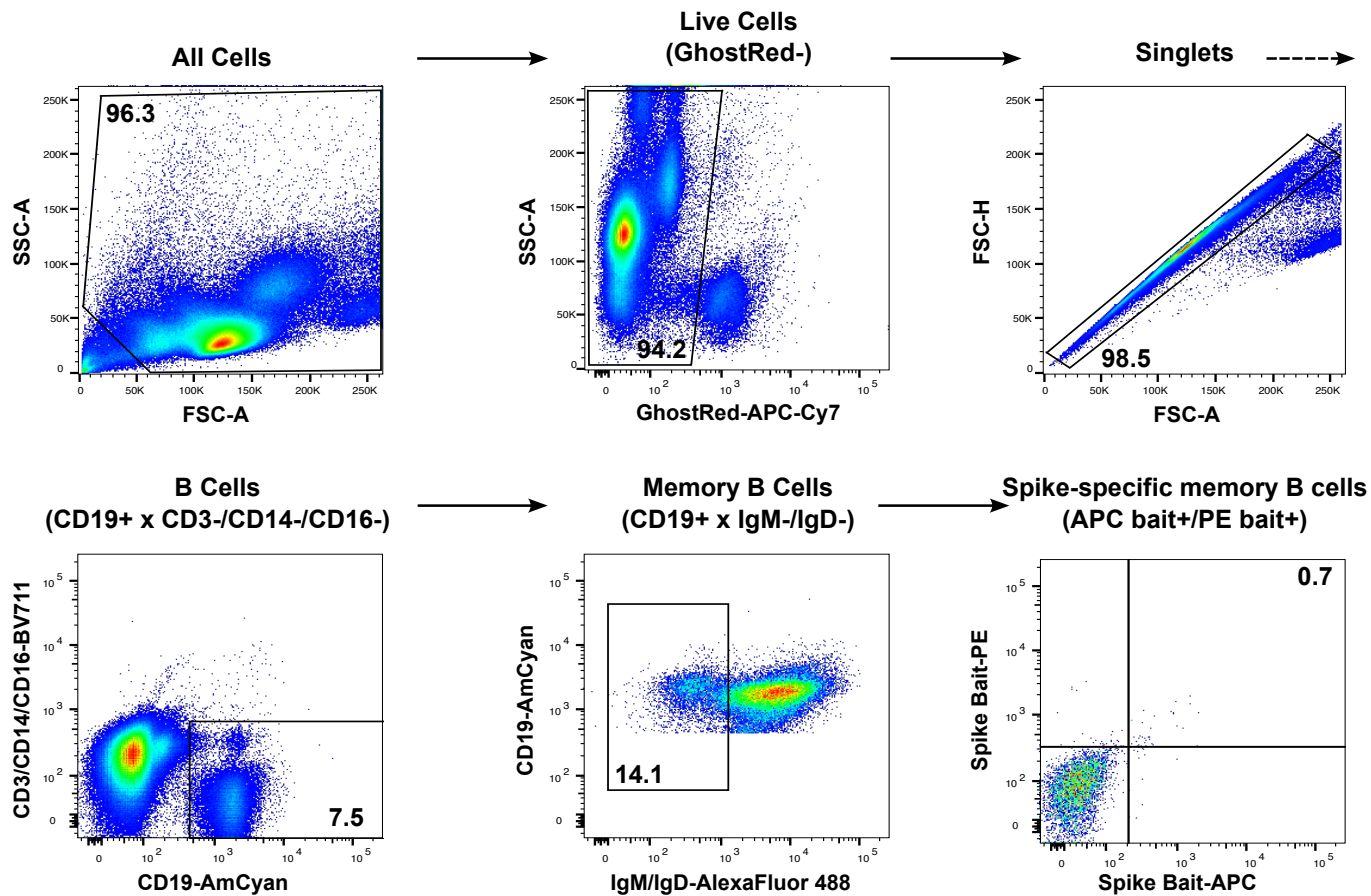

Supplement: S12 Fig — (PDF) [file ppat.1012383.s012.pdf]
