# Supplementary material for: The S2 subunit of spike encodes diverse targets for functional antibody responses to SARS-CoV-2
Source: PLoS Pathog. 2024 Aug 2;20(8):e1012383. doi: 10.1371/journal.ppat.1012383 (PMC11324185; doi:10.1371/journal.ppat.1012383)
Supplement: S5 Fig — Percent competition values are shown for each blocking mAb:biotinylated mAb combination with WH-1 spike trimer (A) or S2 protein (B) as antigen. Values are background-subtracted and averages of two technical replicates. The darker the red, the higher the percent competition. Any negative values were set to zero. (PDF) [file ppat.1012383.s005.pdf]

A

Biotinylated mAb (100ng/mL)

Blocking mAb (2500 ng/mL)

	C68.1	C68.14	C68.16	C68.23	C68.26	C68.35	C68.40	C68.42	C68.43	C68.49	C68.81	C68.93	C68.107	C68.109	C68.144	C68.191	C68.193	C68.204	C68.228	C68.251	C68.265	C68.287	C68.334	C68.337	C68.375	C20.38	C20.59	C20.70	C20.130	C13B8	B6	CV2-2164	CV2-2333
C68.1	98	0	0	2	0	101	0	89	0	0	0	8	0	0	100	0	0	22	0	94	3	99	6	0	100	3	0	101	19	0	0	97	0
C68.4	0	0	0	5	0	0	44	0	0	0	0	0	0	0	0		0	0	0	0	0	0	9	0	0	51	0		0	0	0	26	0
C68.5	2	0	0	1	0	1	17	0	13	0	2	0	0	0	12	14	0	0	6	0	1	0	5	23	4	12	0	26	0	0	0	8	49
C68.14	0	90	10	0	0	86	4	0	0	11	0	87	3	0	95	0	0	0	13	85	6	0	2	0	0	21	0	100	0	0	5	92	0
C68.16	2	0	100	1	0	1	1	1	25	5	1	7	80	0	17	0	3	0	40	2	7	0	2	0	0	53	0	14	38	0	12	0	0
C68.23	0	76	0	60	98	2	0	0	0	100	16	0	59	94	0		36	100	59	0	96	0	98	99	0	0	0		52	0	0	0	0
C68.26	0	12	29	53	100	0	0	0	0	100	0	12	39	97	5	0	0	100	20	9	89	0	100	100	4	28	0	12	36	0	8	0	0
C68.35	54	95	22	1	3	95	43	86	0	0	0	95	27	0	99	13	0	14	10	98	0	2	0	0	96	25	0	100	18	0	1	100	0
C68.40	4	9	4	3	16	4	87	1	0	0	14	0	0	0	0		9	0	4	0	5	6	3	22	0	6	0		6	0	0	1	12
C68.42	98	87	0	0	0	101	2	96	5	0	9	99	0	0	100	0	0	69	0	99	0	100	0	0	99	5	0	101	24	0	2	99	2
C68.43	0	0	1	0	0	0	0	0	65	1	30	19	0	0	8	73	0	13	1	0	3	1	0	0	22	20	0	32	34	0	6	0	75
C68.49	0	0	25	6	58	2	0	1	0	97	9	11	24	41	3	0	0	97	19	0	47	0	81	91	0	31	0	14	40	0	14	0	0
C68.72	2	11	0	0	0	4	0	1	0	53	0	0	0	0	0		4	0	0	0	10	0	5	0	0	0	0		0	0	0	0	0
C68.81	2	14	0	1	0	2	38	1	9	2	77	0	0	0	0		0	0	0	0	2	2	1	0	0	0	0		99	0	0	24	0
C68.93	0	99	28	3	0	99	14	91	0	4	10	99	37	0	99	0	0	10	35	97	10	0	3	26	96	28	0	101	24	0	21	97	12
C68.97	50	91	7	0	0	92	12	71	50	0	0	81	6	0	98	24	4	32	0	93	2	7	0	0	82		0	100	12	0	2	89	10
C68.107	2	8	101	0	0	8	2	0	0	16	10	3	99	0	10	0	49	20	76	2	6	0	2	0	0	69	0	17	34	1	17	0	0
C68.109	2	8	1	65	98	0	0	0	0	100	10	0	36	95	0	0	0	100	5	0	79	0	99	99	0	30	0	3	47	0	9	0	0
C68.144	24	91	0	1	0	91	0	59	10	0	0	89	0	0	97	3	0	6	5	86	2	3	2	0	80	15	0	101	20	0	0	83	0
C68.191	2	0	17	4	0	5	45	0	92	7	0	9	0	0	18	94	0	6	14	0	4	2	7	0	0	30	0	27	33	2	2	48	93
C68.193	1	0	101	0	0	2	20	0	23	7	18	1	100	0	9	28	96	2	99	0	4	1	4	28	0	72	0	7	33	0	11	0	14
C68.204	1	2	26	5	64	10	4	4	0	90	0	14	28	54	10	6	0	97	19	0	56	4	82	90	17	27	0	8	20	2	11	7	6
C68.228	0	0	100	0	0	0	1	0	0	0	9	0	99	0	0		88	0	92	0	0	0	0	0	0		0		39	0	0	0	0
C68.251	90	95	0	1	2	99	0	91	8	0	0	97	11	0	99	10	0	65	0	98	0	16	9	0	99	14	0	101	7	0	0	97	12
C68.265	0	0	62	92	101	0	0	0	0	100	0	2	0	99	4	0	0	100	2	0	97	0	101	100	0	11	7	0	35	0	8	0	0
C68.287	80	0	11	3	0	24	41	0	0	0	0	10	0	0	84	9	0	95	11	3	7	85	7	0	95	28	0	101	23	0	10	95	0
C68.334	2	0	9	55	91	0	15	0	0	99	9	0	27	94	8	0	0	99	28	0	72	1	99	99	3	30	0	7	19	0	11	2	8
C68.337	3	6	0	54	99	2	6	3	0	100	5	0	59	96	0		23	99	55	0	96	6	98	99	0	0	0		37	0	7	0	2
C68.375	75	15	3	0	3	95	0	89	0	5	5	93	25	0	97	5	0	97	20	91	6	84	3	0	97	27	0	101	16	0	6	93	0
C20.36	5	18	0	0	9	2	0	9	0	0	5	2	0	0	0	0	1	0	0	0	10	1	0	0	0	1	0		31	0	2	0	0
C20.38	1	9	16	4	0	0	0	7	0	4	0	3	1	0	11	0	0	1	28	0	0	3	3	0	3	81	0	3	10	0	11	0	0
C20.59	0	5	0	0	0	0	0	0	16	0	17	0	10	0	5	5	2	3	0	0	1	0	0	0	0		52	2	9	1	3	1	0
C20.70	6	35	0	4	0	4	40	10	0	22	0	22	0	0	0	0	0	0	0	0	4	1	0	0	2	0	0		9	0	0	65	0
C20.130	1	5	0	3	0	10	10	0	0	3	59	2	0	1	0		0	0	0	0	3	0	0	41	0	0	0		94	0	0	10	17
C20.174	2	1	0	1	0	3	2	0	0	36	0	0	0	19	0		0	0	0	0	5	0	16	77	0	0	0		0	0	0	0	5
B6	0	18	36	1	0	2	1	2	0	0	13	7	19	0	7	5	0	3	17	0	4	2	2	0	0	23	0	12	28	2	90	0	0
CV2-2002	2	26	2	3	4	1	0	2	36	9	23	15	7	0	4	49	0	2	8	0	1	5	0	0	4	12	0	22	39	0	2	0	55
CV2-2164	58	94	0	2	0	95	0	81	24	0	0	85	0	0	97		0	42	0	83	0	4	0	0	82	53	0	0	9	0	0	85	0
CV2-2333	1	7	0	2	25	3	37	2	80	5	0	5	57	7	0		26	0	53	0	21	2	1	0	0	0	0	0	47	0	3	50	90
CV3-25	1	12	13	0	6	0	16	0	10	8	18	17	8	4	0	0	0	4	5	0	0	0	1	30	6	29	0	4	25	0	71	10	18
76E1	1	4	0	0	0	0	0	0	12	12	0	0	10	0	6	0	0	1	0	1	0	0	2	0	9		0	0	12	9	3	0	1
VP12E7	0	4	0	0	0	0	3	0	8	8	4	4	8	1	1	7	2	2	0	2	2	2	0	1	2		0	0	5	6	2	0	4
C13B8	1	28	4	0	0	1	0	7	33	33	30	30	12	0	2	12	5	7	0	9	3	3	4	3	0		0	0	40	83	2	0	4

B

Biotinylated mAb (1 ug/mL)

Blocking mAb (25 ug/mL)

	C20.38	C20.59	C20.67	C20.119	C20.192	C20.210	C68.16	C68.81	C68.107	C68.204	C68.287	COV2-2164	VP12E7	76E1	C13B8	CV3-25
C20.36	0	1	0	0	0	0	0	0	0	0	0	1	4	2	4	0
C20.38	25	0	98	9	98	1	0	17	1	51	16	6	3	4	5	0
C20.59	0	14	0	3	0	0	0	21	2	47	16	7	3	4	5	0
C20.67	0	0	97	3	48	3	1	1	0	13	11	0	3	0	6	0
C20.82	0	0	0	0	0	0	0	24	1	0	5	1	1	2	3	0
C20.119	0	0	0	86	0	1	0	7	0	15	13	1	8	7	10	0
C20.130	0	0	0	0	2	1	0	72	0	0	0	0	3	1	7	3
C20.174	0	0	0	0	0	0	0	0	2	0	0	0	3	0	2	0
C20.192	0	1	95	0	91	0	0	0	4	12	2	1	4	2	6	0
C20.210	0	0	43	2	0	49	0	0	4	11	14	0	4	4	3	0
C68.1	0	0	0	0	0	0	0	0	6	3	5	0	0	3	3	0
C68.4	1	0	0	0	0	1	0	0	1	0	0	0	0	0	0	2
C68.16	0	0	0	9	0	2	51	0	7	0	0	0	4	2	3	0
C68.72	0	0	0	0	0	0	0	0	1	5	0	3	5	0	6	0
C68.81		0					0		0		0	0	7	0	2	
C68.107	0	0	0	6	0	0	91	0	61	45	0	0	1	8	7	1
C68.204	0	3	0	0	1	0	24	0	6	83	3	0	3	0	2	0
C68.287	0	11	0	0	0	0	0	0	0	36	96	17	4	1	1	0
C68.375	0	0	0	0	0	0	0	0	1	0	4	0	3	0	7	0
COV2-2164	0	0	0	0	1	0	1	0	5	46	56	96	0	0	3	1
VP12E7	0	0	0	91	3	0	0	46	0	55	29	9	63	22	25	0
76E1	0	6	0	97	2	0	0	51	3	64	48	9	82	78	59	2
C13B8	0	69	7	97	0	0	0	39	3	48	32	1	54	38	69	0
COV2-2333	0	0	1	0	0	0	0	0	8	0	0	0	6	0	8	0
CV3-25	2	0	0	0	0	1	0	14	4	60	14	0	0	1	4	51
