# Supplementary material for: The S2 subunit of spike encodes diverse targets for functional antibody responses to SARS-CoV-2
Source: PLoS Pathog. 2024 Aug 2;20(8):e1012383. doi: 10.1371/journal.ppat.1012383 (PMC11324185; doi:10.1371/journal.ppat.1012383)
Supplement: S6 Fig — Also shown are the IGH % SHM (red) and CDR3 aa length (blue) where the darker colors signify a higher value. Genes enriched in a competition group are noted in red. (PDF) [file ppat.1012383.s006.pdf]

Comp Group	mAb ID	IGHV	IGLV or IGKV	Heavy %SHM	Heavy CDR3 length
Group 1	**C68.1**	HV5-10-1	KV4-1	5.0	13
	**C68.14**	HV1-69	KV2-30	4.0	20
	**C68.35**	HV1-69	KV3-11	1.4	15
	**C68.42**	HV3-30	KV1-5	4.0	18
	**C68.93**	HV4-31	KV3-20	3.2	16
	**C68.97**	HV1-69	KV3-11	1.4	15
	**C68.144**	HV1-69	KV3-11	2.2	15
	**C68.251**	HV1-69	KV3-11	3.0	15
	**C68.287**	HV1-46	KV1-5	6.3	14
	**C68.375**	HV3-30	KV3-20	4.6	9
	**C20.70**	HV3-23	LV2-14	2.5	14
Group 2	**C68.23**	HV1-2	KV4-1	2.5	15
	**C68.26**	HV3-30	LV1-47	3.6	13
	**C68.49**	HV3-30	KV1-5	3.6	14
	**C68.109**	HV3-30	KV3-20	3.6	14
	**C68.204**	HV3-21	KV1-NL1	4.6	17
	**C68.265**	HV3-30	KV1D-39	3.6	14
	**C68.334**	HV4-59	KV2D-28	4.7	14
	**C68.337**	HV4-34	KV1D-39	5.9	23
	**C20.174**	HV3-30-3	LV1-40	3.7	11
Group 3	**C68.16**	HV1-46	KV3-20	2.8	13
	**C68.107**	HV3-7	KV1-12	4.1	22
	**C68.193**	HV3-7	LV3-19	1.5	24
	**C68.228**	HV3-7	KV3-15	3.4	22
	**C20.38***	HV3-23	LV8-61	2.8	24
Group 4	**C68.5**	HV1-3	LV3-10	9.7	13
	**C68.40**	HV3-7	LV3-21	2.5	13
	**C68.43**	HV3-74	LV3-1	3.0	14
	**C68.191**	HV3-23	LV3-10	3.7	10
Group 5	**C20.38***	HV3-23	LV8-61	2.8	24
	**C20.67**	HV1-8	KV3-15	0.5	22
	**C20.192**	HV4-34	LV2-14	1.3	18
	**C20.210**	HV3-15	LV2-11	10.0	20
Group 6	**C68.81**	HV3-30	LV3-10	4.1	15
	**C20.59**	HV3-30-3	LV3-1	1.6	15
	**C20.119**	HV3-30-3	LV2-8	12.9	14
	**C20.130**	HV3-30-3	LV3-10	4.9	14
	**C20.36**	HV3-53	KV1-5	0.3	9
	**C20.82**	IGHV4-31	IGKV3-15	0.6	11
	**C68.4**	HV4-59	KV4-1	0.5	21
	**C68.72**	HV1-2	LV1-47	1.3	21
