# Supplementary material for: The S2 subunit of spike encodes diverse targets for functional antibody responses to SARS-CoV-2
Source: PLoS Pathog. 2024 Aug 2;20(8):e1012383. doi: 10.1371/journal.ppat.1012383 (PMC11324185; doi:10.1371/journal.ppat.1012383)
Supplement: S10 Fig — Peptides bound by S2 mAb C68.204 in PhIP-seq (pan-CoV library) are marked with red or blue. The sequence in purple in SARS-CoV-2 is the FP region. Conservation between SARS-CoV-1 and SARS-CoV-2 at each residue is noted under the sequence where stars note identical residues and pink dots notes sites with a different amino acid between the sequences. (PDF) [file ppat.1012383.s010.pdf]

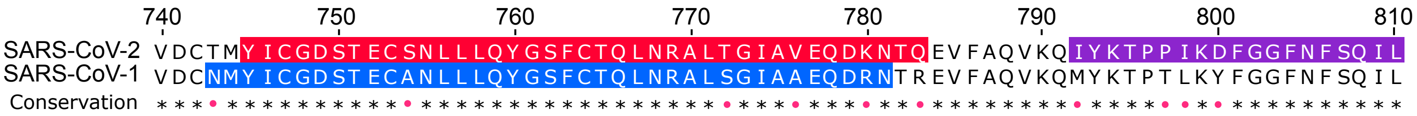
740 750 760 770 780 790 800 810
SARS-CoV-2 VDCTMYICGDSTECSNLLLQYGSFCTQLNRALTGIAVEQDKNTQEVFAQVKQIYKTPPIKDFGGFNFSQIL
SARS-CoV-1 VDCNMYICGDSTECANLLLQYGSFCTQLNRALSGIAAEQDRNTREVFAQVKQMYKTPTLKYFGGFNFSQIL
Conservation ***•**********•*****************•***•***•**•********•****••*•**********
